# A Case Report on a 48-Year-Old Male with Alcohol Use Disorder and Complex Comorbidities

**DOI:** 10.1192/j.eurpsy.2026.10798

**Published:** 2026-07-31

**Authors:** D. Žujić, K. Miljanić

**Affiliations:** 1 University of Zagreb School of Medicine; 2 Health Center of the Ministry of the Internal Affairs, Zagreb, Croatia

## Abstract

**Introduction:**

Alcohol dependence and polysubstance abuse remain among the most difficult psychiatric conditions to treat, particularly when comorbid with severe somatic and psychiatric disorders. This case presents a 48-year-old male, admitted to the emergency psychiatric unit due to acute alcohol intoxication requiring detoxification. Notably, this was his 69th hospitalization within the last two years, reflecting a chronic relapsing course and poor long-term treatment adherence. His medical history is significant for cardiac insufficiency, hiatal hernia, gastropathy with esophageal erosions related to chronic alcohol intake, and chronic post-traumatic stress disorder (PTSD) linked to exposure during the domestic war. This case raises crucial questions about the onset of addiction, treatment resistance, and underlying factors that perpetuate relapse.

**Objectives:**

The objectives were: to stabilize the patient medically and psychiatrically, to analyze reasons for repeated treatment failures despite multiple interventions, and to explore the onset and perpetuation of addiction in the context of early substance use, trauma, and psychiatric comorbidities.

**Methods:**

Following detoxification, the patient was initiated on the following medications: spironolactone 100 mg, buprenorphine 16 mg, diazepam 30 mg daily, quetiapine 25 mg (in the afternoon), quetiapine SR 150 mg, mirtazapine 45 mg (in the evening), and pregabalin 600 mg. He reported prior dependence on heroin (last use 2014), MDMA (2011), and cocaine (2010). Currently, he combines alcohol with benzodiazepines to manage intrusive PTSD symptoms. Importantly, he first used psychoactive substances at age 14, coinciding with the onset of the domestic war.

**Results:**

Detoxification was achieved successfully, and the patient was admitted to psychiatric care for alcohol dependence. Although initially cooperative, he requested discharge after two days, repeating a consistent pattern of premature termination of treatment. This suggests profound treatment resistance and highlights the difficulty of sustaining long-term rehabilitation.

**Conclusions:**

This case illustrates the complexity of addiction as a chronic, multifactorial disorder shaped by genetic predisposition, early exposure, and trauma. The fact that the patient initiated substance use at age 14, before direct war trauma—raises critical questions: when does addiction truly begin? Is it primarily biologically determined, environmentally induced, or trauma-driven? Repeated hospitalizations without sustained engagement emphasize the need for integrated, trauma-informed, and multidisciplinary approaches in treatment. Understanding the timing and roots of addiction remains crucial for designing effective interventions in post-conflict and high-risk populations.

**Disclosure of Interest:**

None Declared

